# Starch and Fiber Contents of Purified Control Diets Differentially Affect Hepatic Lipid Homeostasis and Gut Microbiota Composition

**DOI:** 10.3389/fnut.2022.915082

**Published:** 2022-07-07

**Authors:** Julia Schipke, Christina Brandenberger, Marius Vital, Christian Mühlfeld

**Affiliations:** ^1^Hannover Medical School, Institute of Functional and Applied Anatomy, Hannover, Germany; ^2^Biomedical Research in Endstage and Obstructive Lung Disease Hannover (BREATH), Member of the German Center for Lung Research (DZL), Hannover, Germany; ^3^Hannover Medical School, Institute for Medical Microbiology and Hospital Epidemiology, Hannover, Germany

**Keywords:** diet-induced obesity, mouse model, control condition, purified diets, hepatic steatosis, gut microbiota

## Abstract

**Background:**

Interpretation of results from diet-induced-obesity (DIO) studies critically depends on control conditions. Grain-based chows are optimized for rodent nutrition but do not match the defined composition of purified diets used for DIO, severely limiting the comparability. Purified control diets are recommended but often contain high starch and only minor fiber amounts. It is unknown whether this composition leads to metabolic alterations compared with chow and whether the addition of refined fibers at the expense of starch affects these changes.

**Methods:**

In this experiment, 6-week-old C57BL/6N mice were fed (i) a conventional purified control diet (high-starch, low-fiber; Puri-starch), (ii) an alternative, custom-made purified control diet containing pectin and inulin (medium-starch, higher-fiber; Puri-fiber), or (iii) grain-based chow for 30 weeks (*N* = 8–10).

**Results:**

Puri-starch feeding resulted in significantly elevated levels of plasma insulin (*p* = 0.004), cholesterol (*p* < 0.001), and transaminases (AST *p* = 0.002, ALT *p* = 0.001), hepatic *de novo* lipogenesis and liver steatosis, and an altered gut microbiota composition compared with chow-fed mice. In contrast, Puri-fiber exerted only minor effects on systemic parameters and liver lipid homeostasis, and promoted a distinct gut microbiota composition.

**Conclusion:**

Carbohydrate-rich purified diets trigger a metabolic status possibly masking pathological effects of nutrients under study, restricting its use as control condition. The addition of refined fibers is suited to create purified, yet physiological control diets for DIO research.

## Introduction

Obesity and its related comorbidities, including type 2 diabetes and cardiovascular diseases, affect millions of children and adults worldwide and cause increased mortality rates and soaring healthcare costs (1). This warrants intensive research efforts investigating obesity-related pathological changes and possible intervention strategies. Preclinical animal models provide controlled conditions and a high comparability between the experimental groups and are thus often utilized. Since interpretation of data critically depends on the chosen control conditions, the choice of the control diet is an important issue for animal studies on nutrition-related disorders, that is nevertheless often not addressed adequately (2–4). In normal animal husbandry, mice are fed a grain-based chow, which contains natural ingredients, such as ground corn, wheat and oat, soybean meal and animal by-products, and very high levels of both soluble and insoluble fibers (5). However, its exact composition changes due to harvest supply and is thus charge-dependent (6). Moreover, chow contains various amounts of phytoestrogens that can influence study results (7, 8), restricting its application as control food for diet-induced obesity (DIO) studies. Purified diets have a clearly defined composition, are composed of refined and thus charge-independent ingredients, and are recommended for DIO research (6). However, purified control diets often contain considerable amounts of starch as calorie source, but only less amounts of fibers, which are a major part of physiological mouse food intake. The latter is due to the fact that often cellulose is the only added fiber component that is not palatable and only small amounts are, hence, tolerated by the animals (9). Recently, other purified fiber alternatives, such as pectin from apple or inulin from chicory, are available to increase the fiber content of purified foods. This might constitute a good control diet alternative combining the advantages of a healthy high fiber content and a defined, charge-independent comparable nutrient composition. Therefore, this study aimed at comparing a conventional purified control diet low in fiber and high in starch (Puri-starch), a custom-made purified control diet enriched in fibers and reduced in starch (Puri-fiber) with a usual laboratory, grain-based chow diet (Chow) regarding parameters relevant for DIO research, including systemic alterations, liver lipid homeostasis, and gut microbiota composition.

## Methods

### Mice Husbandry and Diets

**Figure 1:**
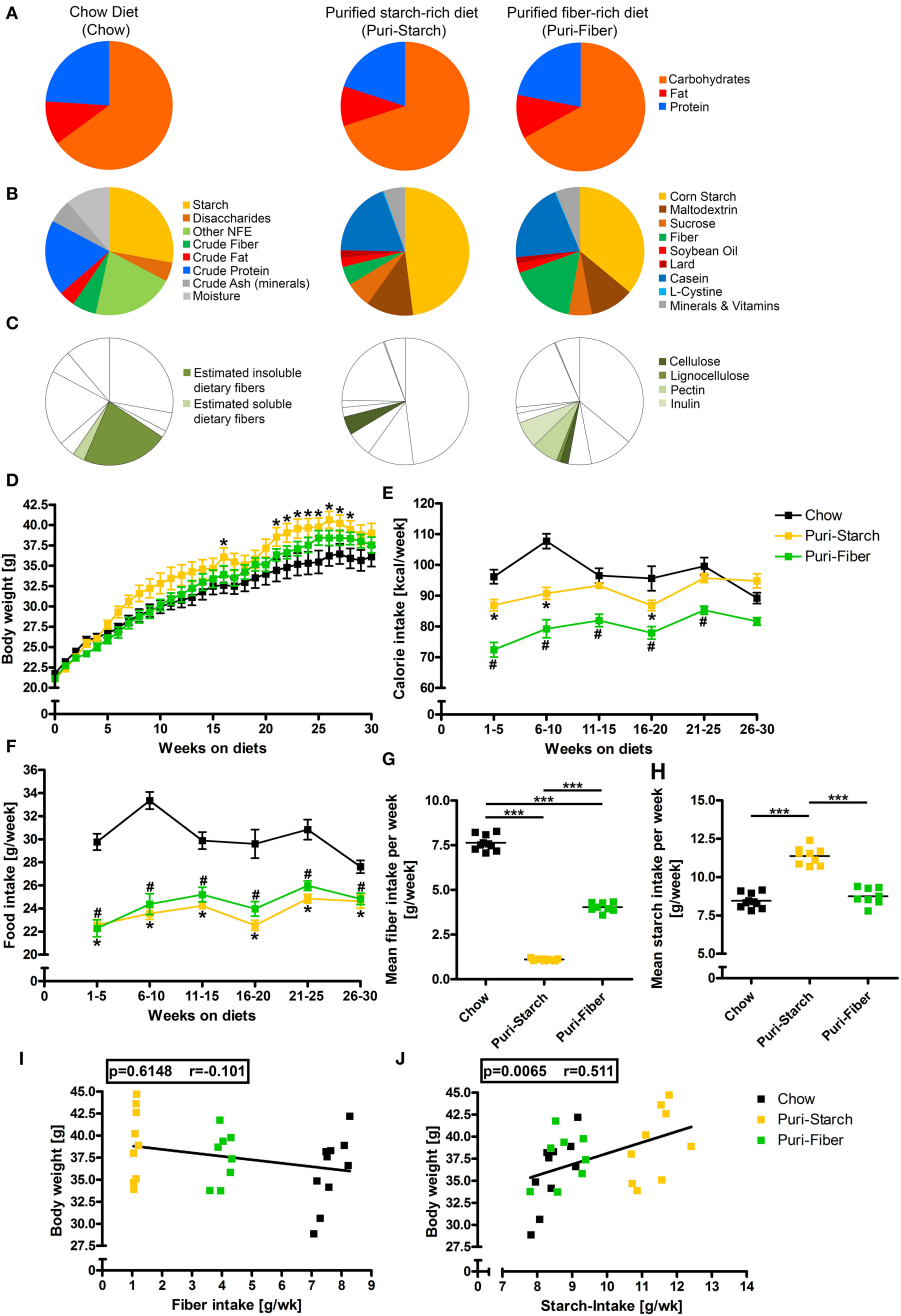
Composition of diets and their effects on body weight, calorie consumption, and food intake. **(A)** Macronutrient composition of diets in kcal%. **(B)** Nutritional composition of diets in g%. **(C)** Fiber content of diets in g%. **(D)** Body weights in g. **(E)** Calorie intake in kcal/week. **(F)** Food intake in g/week. **(G)** Mean fiber intake in g/week. **(H)** Mean starch intake in g/week. **(I)** Correlation of mean fiber intake per week and body weight after 30 weeks. **(J)** Correlation of mean starch intake per week and body weight after 30 weeks. **(D–F)** Data are shown as mean ± SEM, *p < 0.05 Puri-starch compared with Chow, #*p* < 0.05 Puri-fiber compared with Chow (two-way repeated-measures ANOVA and Tukey *post-hoc* tests). **(G,H)** Data are shown as individual data points with indicated means, *p < 0.05, **p < 0.01, *and **p < 0.001 (one-way ANOVA and Tukey *post-hoc* tests). **(I,J)** Data are shown as individual data points with indicated linear regression (*p*-values from Spearman correlation analysis and Pearson correlation coefficient shown). Chow *n* = 10, Puri-starch *n* = 9, and Puri-fiber *n* = 8.

This study was conducted in accordance with German animal protection laws and with the European Directive 2010/63/EU. Animal experiments were approved by the Local Institutional Animal Care and Research Advisory committee and permitted by the Lower Saxony State Office for Consumer Protection and Food Safety (LAVES; file number 13/1244 and 18/2841). Male C57BL/6NCrl mice were purchased from Charles River (Sulzfeld, Germany) at an age of 5 weeks and were randomly allocated to one of 3 study diets after 1 week of acclimatization. Study diets were: (i) grain-based chow diet (1324 TPF, Altromin, Lage, Germany), (ii) purified Puri-starch diet (D12450J, Research diets, New Brunswick, NJ, United States), and (iii) purified Puri-fiber diet (S3542-E040, ssniff Spezialdiäten, Soest, Germany). The composition of the diets is illustrated in Figures 1A–C and is given in Supplementary Table S3. For chow, contents of crude ingredients (i.e., fiber, fat, protein, and ash), moisture, and nitrogen free extractives (NFEs) were provided by the manufacturer. The starch content was determined using a polarimetric method, and the contents of insoluble and soluble dietary fiber were estimated by an enzymatic-gravimetric method. For Puri-starch and Puri-fiber detailed diet compositions were provided by the manufacturer.

Mice had *ad libitum* access to their prescribed diet and drinking water and were housed individually in cages equipped with shelters and nesting material under temperature-controlled conditions (21 ± 2°C). Body weights and calorie intake of some animals in chow and Puri-starch groups were presented previously (10, 11).

### Blood Collection and Plasma Analysis

After 10, 20, and 30 weeks, mice were fasted for 6 h, retro-orbital blood was collected, and blood plasma was isolated by centrifugation. Plasma concentrations of lipids, enzymes, and glucose were analyzed in the clinical chemistry department of the Hannover Medical School using kits according to the manufacturer's instructions (Roche Diagnostics, Mannheim, Germany) and automated analyzer systems (Roche Diagnostics, Mannheim, Germany). Insulin levels were assessed in duplicates by an ultrasensitive-mouse-insulin-ELISA (#90080, Chrystal Chem, Elk Grove Village, IL, United States). HOMA-IR values were calculated as follows: Glucose (mmol/L) × insulin (μU/ml) / 22.5. Moreover, 1/fasting insulin was calculated as surrogate index for insulin sensitivity. Since adjustment to weight was shown before to considerably enhance correlation of these surrogate indexes with glucose clamp-derived measures (12), HOMA-IR, and 1/fasting insulin are given as body weight ratios.

### Oral Glucose Tolerance Test

After 29 weeks, mice were feed-deprived for 6 h, a baseline blood sample was collected from the tail vein followed by oral gavage of 1 mg glucose/g body weight. Furthermore, blood samples were obtained from the tail after 15, 30, 60, and 120 min. Blood glucose was assessed with a glucometer (Wellion Calla; Med Trust). The area under the glucose tolerance curve (area under the curve (AUC) was calculated using the trapezoidal rule.

### Necropsy and Sample Preparation

After 30 weeks, mice were killed under deep anesthesia induced by intraperitoneal injection of ketamine (100 mg/kg body weight) and xylazine (5 mg/kg body weight) by exsanguination. Livers were isolated and cut into ~1 mm slices, which were randomly assigned to either freezing in liquid nitrogen and storage at −80°C or to fixation in 4% paraformaldehyde/0.2 M Hepes buffer (pH 7.4) for at least 24 h. From the latter, random 1 mm × 1 mm × 1 mm blocks were cut, incubated in 1.5% glutaraldehyde/1.5% paraformaldehyde/0.15 M Hepes buffer (pH 7.4) for at least 24 h, postfixed in 1% osmium tetroxide in 0.1 M sodium cacodylate (pH 7.4), stained *en bloc* with half-saturated uranyl acetate in water, and dehydrated in an ascending acetone series before final embedding in epoxy resin according to the manufacturer's instructions.

Feces samples were collected from the cecum and the distal colon, snap-frozen in liquid nitrogen, and stored at −80°C.

### Liver Histology

From 3 epoxy resin blocks per animal, 1 μm thick sections were cut, mounted on glass slides, and stained with toluidine blue. The sections were digitalized with a histological slide scanner (AxioScan.Z1; Zeiss, Oberkochen, Germany) at an objective lens magnification of 40×. Automated image analysis was performed using the tissuemorph DP software (Visopharm, Horsholm, Denmark). An application was designed for threshold-based detection of lipid droplets in toluidine blue stained liver tissue. First, a multistep gray scale conversion was performed for foregrounding the signal of the lipid droplets. This was carried out using the blue color band (RGB-B) and the chromaticity blue–converted version of the image. The signal of the two image bands was multiplied and then square root transformed. In a next step, a threshold of 0 to 3,500–7,000 (depending on the staining intensity) was applied to select the area of lipid droplets within the liver tissue. The percentage of the lipid fraction was finally calculated by dividing the area of lipid droplets by the total area of the liver section.

### Liver mRNA Expression Analysis

mRNA was isolated from liver samples using the NucleoSpin RNA/Protein Kit (#740933.250, Macherey-Nagel, Düren, Germany) according to the manufacturer's manual. RNA concentration was measured with a NanoDrop 2,000 Spectrophotometer (Thermo Fisher Scientific, Waltham, MA, USA), and cDNA was generated by transcription of 1 μg RNA using the iScript cDNA Synthesis Kit (BioRad, Hercules, CA, USA) according to the manufacturer's instructions. Real-time PCR reactions were performed with a C1000 Thermal Cycler (CFX384 Real-Time System, BioRad) utilizing fluorescein amidite (FAM)-labeled primers and the iTaq Universal Probes Supermix (BioRad). The following primers were used: ACLY, qMmuCEP0053217; ACCα (Acaca), qMmuCIP0030034; ACCβ (Acacb), qMmuCIP0030144; FAS (Fasn), qMmuCEP0054102; SCD1, qMmuCIP0031297; FABP1, qMmuCIP0034032; SCP2, qMmuCEP0056614; GPAT1, qMmuCIP0034231; Lipin1 (Lpin1), qMmuCIP0031637; Lipin2 (Lpin2), qMmuCIP0032366; DGAT2, qMmuCIP0030922; CCTα (PCYT1a), qMmuCIP0031233; PLIN2, qMmuCIP0033479; ATGL (Pnpla2), qMmuCEP0034900; CPT1a, qMmuCEP0054021; MTPα (Hadha), qMmuCEP0054151; MTPβ (Hadhb), qMmuCIP0062992; SREBP1 (Srebf1), qMmuCIP0033121; SREBP2 (Srebf2), qMmuCIP0035147; PPARα (Ppara), qMmuCEP0054952 (BioRad). The thermo cyclic protocol was as follows: initially 2 × 95°C for 2 min, followed by 40 cycles of 95°C for 5 s and 60°C for 20 s. For each target, all samples to be compared were run in parallel on the same 384-well plate. The samples were analyzed as triplicates. The relative mRNA expression of a gene of interest was assessed by normalization to HPRT as housekeeping gene (ΔCt) and to the chow-fed animals as “control group” (ΔΔCt), finally the fold expression was calculated (2^ΔΔCt^).

### Microbiome Analysis

Fecal DNA was extracted using the ZymoBIOMICS DNA Kit according to the manufacturer's instructions (Zymo Research, Irvine, CA, USA). Amplification of the V3V4 region of the 16S rRNA-gene was carried out using a two-step PCR, and sequencing was performed on Illumina MiSeq (2x250)(13). Obtained sequences were processed using the DADA2 pipeline in R (14), where amplicon sequence variants (ASVs) were assigned the RDP taxonomy. Follow-up analyses were carried out within phyloseq (15) on relative abundance data (**Figure 4** and Supplementary Tables S1, S2); for diversity measures, samples were rarefied to equal depth (4,700 sequences). Ordination was based on principal coordinate analyses using Bray-Curtis dissimilarities of proportional count data.

### Statistical Analysis

Statistical parameters are stated in the specific figure legends. Two-way repeated-measures ANOVA, followed by a *post-hoc* Tukey test, and one-way ANOVA and a *post-hoc* Tukey test were performed using Sigma Plot version 13.0 (Systat Software Inc.). Spearman correlation analysis and calculation of Pearson correlation coefficient were performed using GraphPad Prism (version 4; GraphPad Software). PERMANOVA analyses of microbiota composition were performed in R (function adonis from the vegan package), and differential abundance analysis of microbial taxa was carried out based on Analysis of Compositions if Microbiomes with Bias Correction (ANCOM-BC) (16). *P*-values < 0.05 were considered statistically significant. Data are presented as dot plots with individual values and indicated means or as means ± SEM. All n values are true biological replicates (separate mice). Figures were created with Adobe Photoshop (Version 13.0).

## Results and Discussion

### Diet Composition Affects Food Intake and Body Weight

The analyzed diets were similar in macronutrient contents (Figure 1A, Supplementary Table S3) but differed substantially in their detailed composition with starch accounting for ~28 g% in Chow, 48 g% in the Puri-starch diet, and 36 g% in the Puri-fiber diet (Figure 1B, Supplementary Table S3). While Chow contained ~25 g% soluble and insoluble fibers, the Puri-starch diet contained 4.7 g% cellulose representing the whole fiber amount of this diet. In contrast, the Puri-fiber food contained 2 g% cellulose, 1 g% lignocellulose, 6.8 g% pectin, and 6.8 g% inulin, which together accounted for 16.6 g% fiber content (Figure 1C, Supplementary Table S3).

The Puri-starch-fed mice were heavier compared with Chow-fed mice from week 21 to 28, whereas the body weight of Puri-fiber-fed mice did not differ significantly from Chow-fed mice at any time point during the protocol (Figure 1D). Chow-fed mice ingested significantly higher amounts of food and calories compared with both purified diets (Figures 1E,F). Puri-fiber-fed mice ingested the lowest amount of calories of all groups what may be related to the added dietary fibers pectin and inulin that were shown previously to decrease food intake, body weight, and adiposity (17).

Chow-fed mice consumed the highest mean fiber amount per week, whereas Puri-fiber-fed mice ingested less fibers and Puri-starch-fed animals only minor fiber amounts (Figure 1G). Mice in the Puri-starch group had a significantly higher mean starch intake compared with both Chow- and Puri-fiber-fed mice (Figure 1H). Moreover, the mean starch intake correlated with the body weight after 30 weeks (Figure 1J), which was not the case for the fiber intake (Figure 1I).

In summary, starch content and fiber composition of the individual diets affected both food intake and body weight. A high starch intake was associated with higher body weights.

### Puri-Starch Diet Increases Plasma Insulin, Cholesterol, and Transaminase Levels

**Figure 2:**
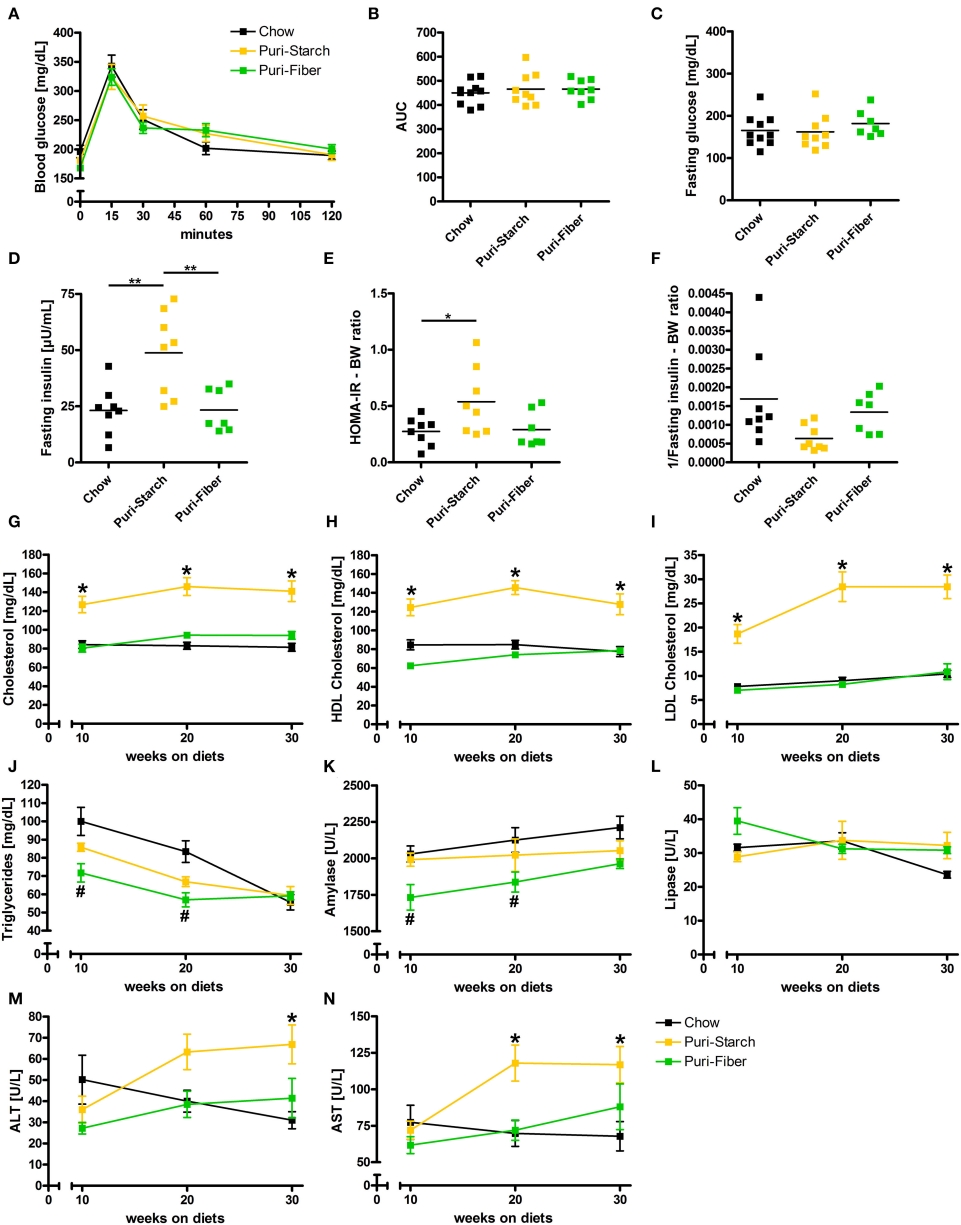
Effects of diets on glucose homeostasis and circulating lipids and enzymes. **(A)** Blood glucose concentrations during the oral glucose tolerance test. **(B)** Area under the curve (AUC) of glucose tolerance test curves. **(C)** Fasting plasma glucose concentrations. **(D)** Fasting plasma insulin concentrations. **(E)** Calculated Homeostatic Model Assessment for Insulin Resistance (HOMA-IR) related to body weight. **(F)** Calculated 1/insulin (index for insulin sensitivity) related to body weight. **(G)** Fasting plasma total cholesterol concentrations. **(H)** Fasting plasma HDL cholesterol concentrations. **(I)** Fasting plasma LDL cholesterol concentrations. **(J)** Fasting plasma triglycerides concentrations. **(K)** Fasting plasma amylase concentrations. **(L)** Fasting plasma lipase concentrations. **(M)** Fasting plasma alanine transaminase concentrations. **(N)** Fasting plasma aspartate transaminase concentrations. **(A,G–N)** Data are shown as mean ± SEM, *p < 0.05 Puri-starch compared with Chow, #*p* < 0.05 Puri-fiber compared with Chow (two-way repeated-measures ANOVA and Tukey *post-hoc* tests). **(B–F)** Data are shown as individual data points with indicated means, *p < 0.05, **p < 0.01, and ***p < 0.001 (one-way ANOVA and Tukey *post-hoc* tests). Chow *n* = 10, Puri-starch *n* = 9, and Puri-fiber *n* = 7–8.

In DIO research, it is important that the control diet does not have a major influence on glucose and fat metabolism. The glucose tolerance and the fasting blood glucose concentration were not affected by any of the diets (Figures 2A–C). In contrast, fasting insulin levels and HOMA insulin resistance related to body weight were increased in Puri-starch-fed animals compared with Chow and Puri-fiber or compared to Chow, respectively (Figures 2D,E). This is in accordance with previous reports using Puri-starch as control diet for 12 weeks in C57BL/6J mice (18) and for 19 weeks in Sprague-Dawley rats (19). In contrast, other studies using shorter feeding periods (8–9 weeks) observed lower and Chow-like insulin concentrations (20–22), indicating feeding duration-related effects. Moreover, the insulin sensitivity related to body weight showed a strong tendency to be decreased in Puri-starch compared with chow (*p* = 0.052).

Circulating total cholesterol, high-density lipoprotein (HDL) cholesterol and low-density lipoprotein (LDL) cholesterol concentrations were significantly higher under the Puri-starch regimen compared with Chow during the whole course of the experiment (Figures 2G–I). Similar cholesterol levels were observed before in several studies that applied the Puri-starch diet as control condition (19, 23–25). Moreover, alanine transaminase (ALT) and aspartate transaminase (AST) were increased after 30 or after 20 and 30 weeks, respectively (Figures 2M,N), indicating hepatocyte injury. In contrast, plasma cholesterol and transaminase levels of Puri-fiber-fed mice were similar to the Chow group (Figures 2G–I,M,N).

In conclusion, Puri-starch diet caused a prediabetic metabolic condition and had significant adverse effects on circulating blood lipids. In contrast, Puri-fiber diet had no negative effects on glucose and lipid parameters and showed similar values as Chow.

### Puri-Starch Diet Causes Hepatic Lipid Accumulation due to de novo Lipogenesis

**Figure 3:**
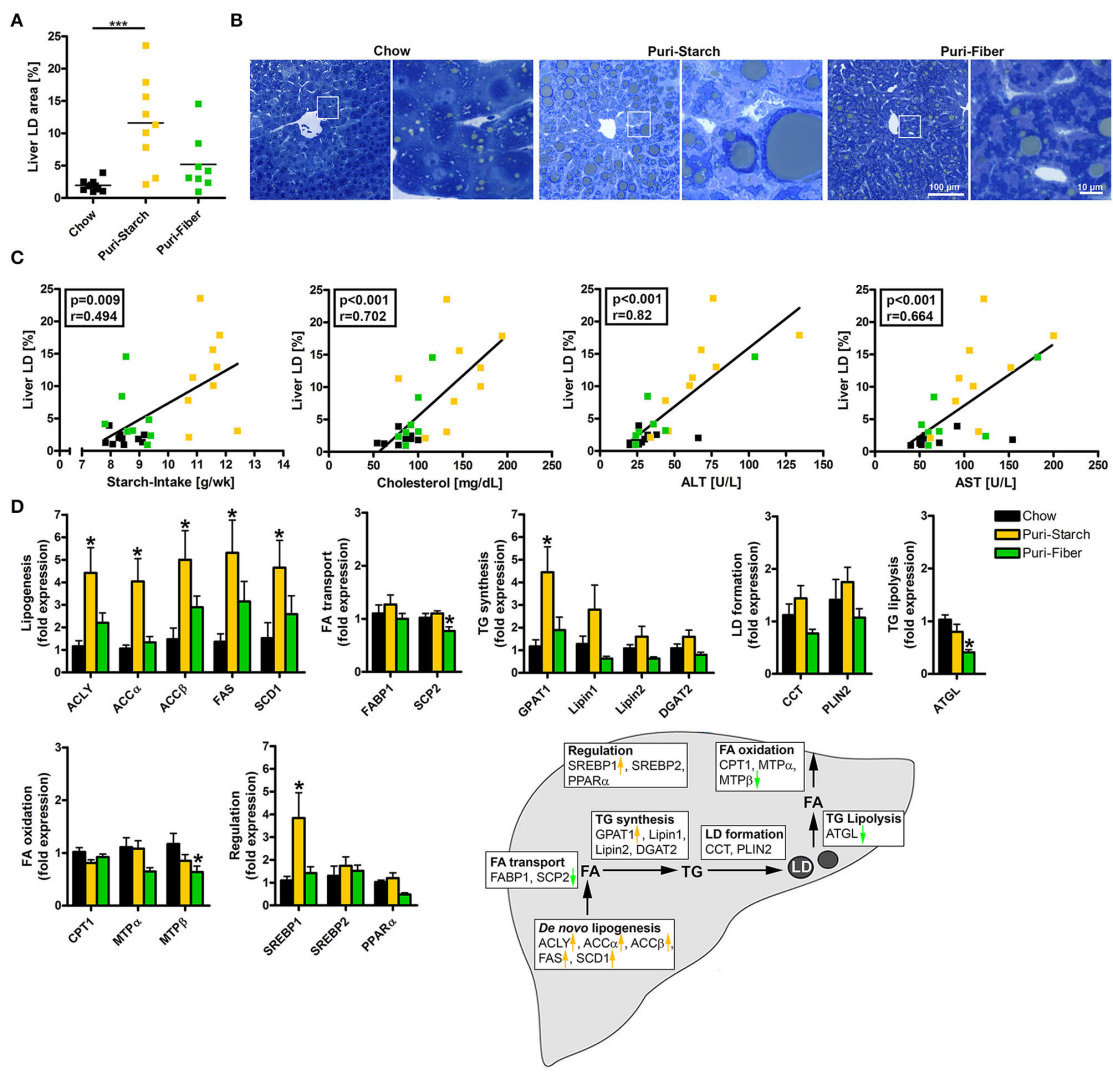
Effects of diets on hepatic lipid accumulation and liver lipid metabolism. **(A)** Percentage of area occupied by lipid droplets in the liver. Data are shown as individual data points with indicated means, ***p < 0.001 (one-way ANOVA and Tukey *post-hoc* test). **(B)** Representative images of liver sections stained with toluidine blue, enlargements of indicated regions shown next to the overviews. **(C)** Correlations of liver lipid droplet area and (i) mean starch-intake averaged over 30 weeks, (ii) plasma cholesterol concentration after 30 weeks, (iii) plasma ALT concentration after 30 weeks, and (iv) plasma AST concentration after 30 weeks. Data are shown as individual data points with indicated linear regression (*p*-values from Spearman correlation analysis and Pearson correlation coefficient given in rectangles). Chow *n* = 10, Puri-starch *n* = 9, and Puri-fiber *n* = 8. **(D)** mRNA expression levels of proteins implicated in hepatic liver metabolism. Data are shown as mean ± SEM, *p < 0.05 compared with Chow (one-way ANOVA and Tukey *post-hoc* test). Chow *n* = 8, Puri-starch *n* = 9, and Puri-fiber *n* = 8.

The increases in ALT and AST in Puri-starch-fed animals were accompanied by a profound hepatic lipid accumulation (Figures 3A,B). The area of lipid droplets in the liver positively correlated with the mean starch intake of the animals over 30 weeks, as well as the circulating cholesterol, ALT and AST concentrations after 30 weeks (Figure 3C). A lipogenic effect of Puri-starch on the liver was also mentioned by two recent reports using the diet as control condition (26, 27).

In the liver, the Puri-starch diet induced the expression of the lipogenic transcription factor SREBP1 and of all analyzed enzymes involved in *de novo* lipogenesis. With the exception of glycerol-3-phosphate acyltransferase (GPAT 1), Puri-starch had no further effects on the analyzed hepatic lipid metabolism enzymes. This is in accordance with previous studies from rodents and humans, demonstrating that excess carbohydrate intake stimulates the hepatic *de novo* lipogenesis resulting in increased intrahepatic triglyceride content (28). In contrast, the Puri-fiber diet increased the liver lipid content only slightly (not reaching statistical significance) and had no significant effect on any of the lipid metabolic pathways except a decreased expression of single enzymes involved in triglyceride lysis and fatty acid oxidation (Figures 3A,B,D). However, expression of lipogenic enzymes showed a tendency to increased levels in Puri-fiber compared with Chow (Figure 3D), despite similar starch intake (Figure 1H). This indicates that the lipogenic response to the purified diets is not simply a starch-related effect but may also be associated with the contained fiber types as well. The dietary fibers pectin and inulin in the Puri-fiber diet are indigestible by mice but are fermented by gut microbiota to produce short chain fatty acids (SCFAs) (29). SCFAs in turn can be used as fuel source or act as signaling molecules excerting regulatory functions in local, intermediary, and peripheral metabolism (30, 31). Moreover, dietary fiber supplementation leads to overall shifts of microbiota composition (32, 33), which was analyzed in a next step.

### Purified Diets Change Gut Microbiota Composition

**Figure 4:**
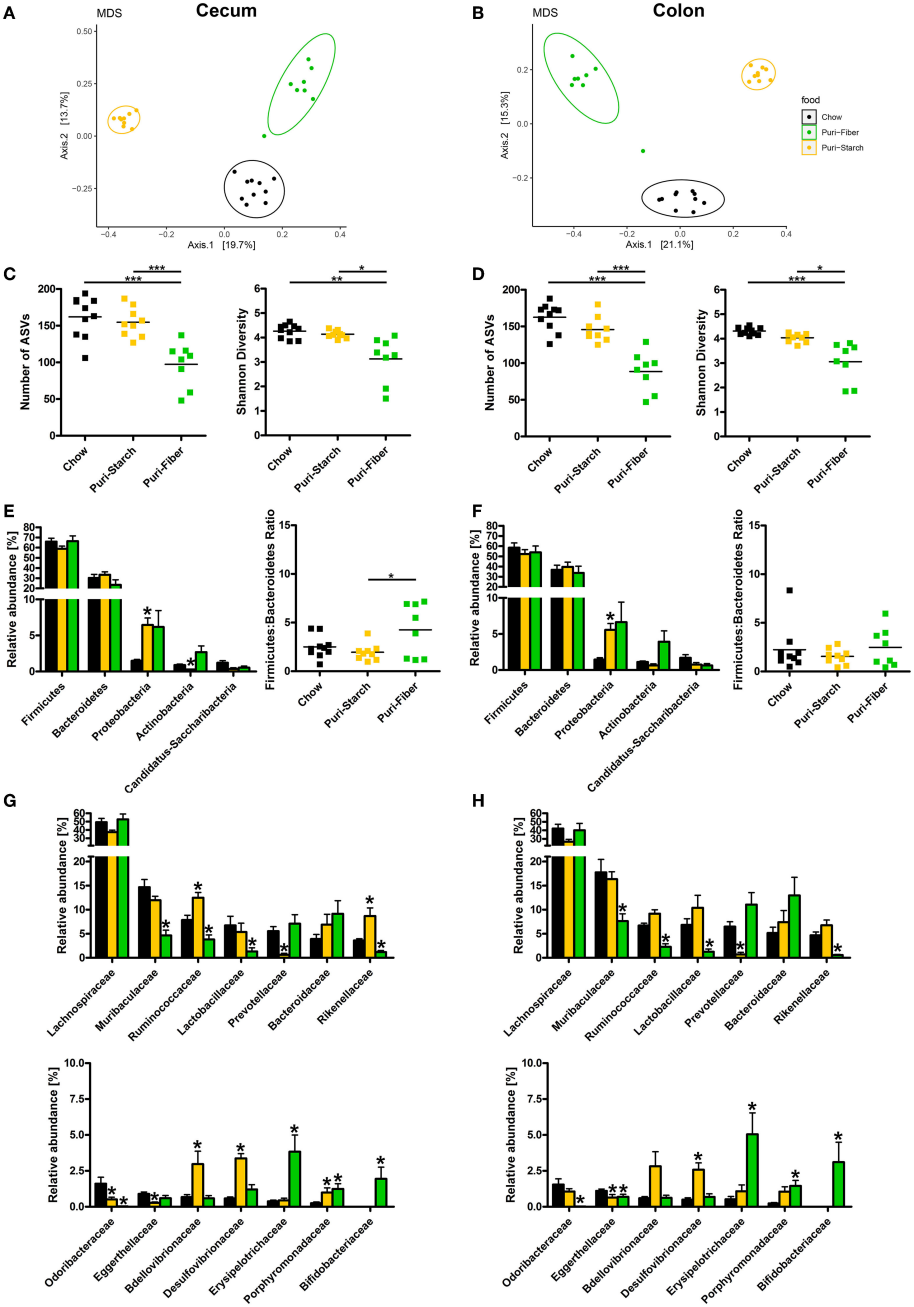
Effects of diets on gut microbiota composition. **(A,B)** Microbial community composition of individual mice shown by multidimensional scaling analysis (principal coordinates based on Bray-Curtis dissimilarities). **(C,D)** Microbiota diversity; data are shown as individual data points with indicated means, *p < 0.05, **p < 0.01, and ***p < 0.001 (one-way ANOVA and Tukey *post-hoc* tests). **(E,F)** Phyla relative abundances; data are shown as mean ± SEM, *p < 0.05 compared with Chow (ANCOM-BC analysis); Firmicutes-to-Bacteroidetes ratio shown as individual data points and indicated means, *p < 0.05 (one-way ANOVA and Tukey *post-hoc* tests). **(G,H)** Family relative abundances; data are shown as mean ± SEM, *p < 0.05 compared with Chow (ANCOM-BC analysis). **(A,C,E,G)** analysis of cecum luminal contents; **(B,D,F,H)** analysis of colon luminal contents. Chow *n* = 10, Puri-starch *n* = 9, and Puri-fiber *n* = 8.

The gut microbiota composition is susceptible to nutritional changes and is believed to play a causal role in the development of obesity and its comorbidities, making it an emerging field for DIO studies. Each of the tested diets was associated with a distinct microbiota composition that clustered separately from the other groups (Figures 4A,B). Moreover, permutational ANOVA analyses using Bray-Curtis dissimilarities of normalized abundance data indicated that bacterial composition between the groups (beta-diversity) was significantly different (*p* < 0.01). The microbial diversity, reflected by the number of observed ASVs and the Shannon Diversity Index, was similar in Chow- and Puri-starch-fed mice but diminished under the Puri-fiber regimen in both the cecum and colon (Figures 4C,D). Thus, the high intake of inulin and pectin in the Puri-fiber group possibly promoted the growth of specific bacteria specialized for the degradation of these fiber types, whereas the complex fibers in chow or the high starch amount in Puri-starch exerted less selective pressure. Interestingly, reduced diversity was also observed in humans on high-fiber diets, which was associated with alleviating type-2 diabetes (34). The authors argued that administration of fibers promoted selective growth of specific SCFA-producing bacteria that resulted in reduced overall diversities, challenging the common view that higher diversity is per se beneficial for the host.

Differential abundance analyses of taxa showed increases in Proteobacteria in the cecum and colon of Puri-starch-fed mice compared with the Chow group, representing major indications of dysbiosis (35), which was accompanied by a decrease in Actinobacteria (cecum only) (Figures 4E,F). The Firmicutes:Bacteroidetes ratio was slightly increased in the cecum of Puri-fiber-fed animals (Figure 4E) supporting its role in fiber degradation (36). At the family level, several differences between purified diets and Chow were detected at both sites (Figures 4G,H). For instance, the abundance of Prevotellaceae that was shown to be associated with human body mass index (BMI) (37) and believed to promote beneficial effects on glucose metabolism (38) was reduced in the Puri-starch group. In contrast, Desulfovibrionaceae were increased—a sulfate-reducing bacterial family that produces hydrogen sulfide that is known to elicit adverse effects (39). In Puri-fiber fed mice, the abundance of Bifidobacteriaceae, which are SCFA-producing bacteria implicated in pectin and inulin fermentation (29) was increased, whereas Prevotellaceae were reduced.

As a limitation of this study, energy expenditure was not measured. It was shown for nutritional components, such as soy-derived phytoestrogens, that they can affect energy expenditure in mice (8). Thus, it is possible that diet-related differences in energy expenditure contributed to the findings reported in this study, and this parameter should be addressed in future studies. Besides diet, housing conditions can influence experimental results from animal studies. It is increasingly acknowledged that standard housing temperatures of 21°C are below the thermoneutrality of mice (which is about 30°C). This mild cold stress can influence various physiological parameters, including sympathetic activity, heart rate, and energy expenditure (40). Since in this study, the housing temperature (21°C) was the same for all mice, it is unlikely that it influenced the reported results. However, environmental temperatures should be taken into account for future studies to increase the translation of findings in animal models to insights into human disease.

## Conclusion

The choice of control diet composition is of high importance to animal studies on nutrition-related disorders. This study demonstrates that despite similar macronutrient composition, grain-based chow, and purified control diets differ significantly regarding their effects on circulating insulin and lipids, liver lipid homeostasis, and gut microbiota. A starch-rich purified diet induced higher body weights, higher plasma insulin, cholesterol and transaminase concentrations, liver steatosis, and elevated hepatic expression levels of enzymes involved in *de novo* lipogenesis compared with chow. This was accompanied by alterations in gut microbiota composition that was characterized by an increase in potentially harmful bacteria. In contrast, a fiber-rich-purified diet resulted in lower calorie intake, normalization of body weight, plasma insulin and cholesterol, and only minor alterations in liver lipid homeostasis. The gut microbiota displayed unique signatures indicative of fiber degradation.

Thus, purified control diets containing high carbohydrate amounts lead to metabolic alterations themselves that presumably mask the pathological effects of nutrients under study, restricting its use as control condition. Grain-based chow consists of complex varying natural sources that do not match the composition of purified experimental diets used for DIO, limiting the comparability and rendering it suboptimal as control diet. The addition of refined fibers at the expense of carbohydrates is suitable to create purified control diets that combine a defined composition matching the experimental diet under study, as well as a physiological metabolic status, and are yet well suited as control condition for DIO studies.

## Data Availability Statement

The original mRNA expression data presented in the study are publicly available. This data can be found here: https://doi.org/10.6084/m9.figshare.20131796.

## Ethics Statement

The animal study was reviewed and approved by the Local Institutional Animal Care and Research Advisory Committee and the Lower Saxony State Office for Consumer Protection and Food Safety (LAVES; File Number13/1244 and 18/2841).

## Author Contributions

JS was responsible for conception and design of the study and prepared figures. JS, CB, and MV performed experiments. JS, CB, MV, and CM analyzed data and approved the final version of the manuscript. JS and CM interpreted results of experiments and drafted the manuscript. CB and MV revised the manuscript. All authors contributed to the article and approved the submitted version.

## Funding

JS and CM acknowledge funding from the Doktor Robert Pfleger-Stiftung. JS was funded by the Else Kröner-Fresenius Stiftung (2019_A77). MV was funded by the Deutsche Forschungsgemeinschaft (#456214861).

## Conflict of Interest

The authors declare that the research was conducted in the absence of any commercial or financial relationships that could be construed as a potential conflict of interest.

## Publisher's Note

All claims expressed in this article are solely those of the authors and do not necessarily represent those of their affiliated organizations, or those of the publisher, the editors and the reviewers. Any product that may be evaluated in this article, or claim that may be made by its manufacturer, is not guaranteed or endorsed by the publisher.
