# Supplementary material for: Starch and Fiber Contents of Purified Control Diets Differentially Affect Hepatic Lipid Homeostasis and Gut Microbiota Composition
Source: Front Nutr. 2022 Jul 7;9:915082. doi: 10.3389/fnut.2022.915082 (PMC9301012; doi:10.3389/fnut.2022.915082)
Supplement: Supplementary file 1 [file Data_Sheet_1.PDF]

Supplementary Material

for the article

Starch and fiber contents of purified control diets differentially affect hepatic lipid homeostasis and gut microbiota composition

by

Julia Schipke, Christina Brandenberger, Marius Vital and Christian Mühlfeld

Supplementary Table S1. Genus abundances (given in means).

	**Cecum**			**Distal Colon**		
	**Chow**	**Puri-Starch**	**Puri-Fiber**	**Chow**	**Puri-Starch**	**Puri-Fiber**
Acetatifactor	2.0	8.7	0.3	2.1	5.9	0.2
Adlercreutzia	0.5	0.2	0.6	0.6	0.6	0.7
Alistipes	3.6	8.6	1.2	4.7	6.7	0.6
Bacteroides	3.8	6.9	8.4	5.1	7.4	12.3
Bifidobacterium	0.0	0.0	1.9	0.0	0.0	3.1
Clostridium_XlVa	2.0	5.9	5.6	2.0	4.3	4.5
Desulfovibrio	0.6	3.4	1.2	0.5	2.6	0.7
Dorea	0.5	0.3	1.2	0.4	0.2	0.8
Duncaniella	7.4	1.7	2.4	9.6	2.2	3.8
Eisenbergiella	2.0	0.0	1.8	1.3	0.0	0.1
Flintibacter	1.3	3.1	0.8	1.0	1.6	0.4
Intestinimonas	0.4	1.7	0.3	0.5	1.7	0.3
Kineothrix	5.7	3.9	0.5	4.4	2.8	0.3
Klebsiella	0.0	0.0	3.9	0.0	0.0	4.7
Lacrimispora	2.0	0.0	0.0	1.2	0.0	0.0
Lawsonibacter	0.5	1.5	0.4	0.4	1.2	0.1
Ligilactobacillus	5.8	4.2	1.2	6.3	9.3	1.1
Longibaculum	0.1	0.0	2.0	0.2	0.0	2.2
Muribaculum	2.8	2.6	0.8	3.1	3.6	1.3
Odoribacter	1.6	0.5	0.0	1.5	1.1	0.0
Oscillibacter	1.6	2.1	0.3	1.2	1.1	0.1
Parabacteroides	0.2	1.0	1.2	0.2	1.1	1.5
Paramuribaculum	2.8	1.3	0.2	2.7	1.7	0.3
Prevotella	4.2	0.0	6.5	5.1	0.0	10.5
Prevotellamassilia	1.3	0.6	0.0	1.3	0.7	0.0
Schaedlerella	0.9	0.7	1.9	0.6	0.4	1.3
Sellimonas	0.0	1.9	0.0	0.0	1.4	0.0
Vampirovibrio	0.7	3.0	0.6	0.6	2.8	0.6

Supplementary Table S2. Group differences related to genus abundances (*p<0.05, ANCOM-BC analysis)**.**

	**Cecum**			**Distal Colon**		
	**Puri-Fiber vs. Chow**	**Puri-Starch vs Chow**	**Puri-Starch vs Puri-Fiber**	**Puri-Fiber vs. Chow**	**Puri-Starch vs Chow**	**Puri-Starch vs Puri-Fiber**
Acetatifactor	*	*	*	*	*	*
Adlercreutzia						
Alistipes	*	*	*	*		*
Bacteroides						
Bifidobacterium	*		*	*		*
Clostridium_XlVa		*			*	
Desulfovibrio		*	*		*	*
Dorea						
Duncaniella	*	*		*	*	
Eisenbergiella		*			*	
Flintibacter		*	*	*		*
Intestinimonas	*	*	*	*	*	*
Kineothrix	*		*	*		*
Klebsiella						
Lacrimispora	*	*		*	*	
Lawsonibacter	*		*	*	*	*
Ligilactobacillus	*		*	*		*
Longibaculum	*		*			*
Muribaculum	*		*			*
Odoribacter	*	*	*	*		*
Oscillibacter	*		*	*		*
Parabacteroides	*	*		*		
Paramuribaculum	*		*	*		*
Prevotella		*	*		*	*
Prevotellamassilia	*		*	*		*
Schaedlerella						
Sellimonas		*	*		*	*
Vampirovibrio		*	*		*	*

Supplementary Table S3. Diet composition.

**Grain-based**		**Purified**		
	**Chow**		**Puri-Starch**	**Puri-Fiber**
[kcal%]		[kcal%]		
Carbohydrates	65	Carbohydrates	70	67
Fat	11	Fat	10	11
Protein	24	Protein	20	22
[g%]		[g%]		
Starch	27.9	Corn Starch	48	36
Disaccharides	4.8	Maltodextrin	11.9	11
Other nitrogen-free extract materials	20.7	Sucrose	6.5	5.9
Crude Fiber	6.1	Fiber	4.7	16.6
Crude Fat	4.1	soybean oil	2.4	2.4
Protein	19.2	Lard	1.9	1.6
Crude Ash (minerals)	5.9	Casein	19	20
Moisture	11.3	L-Cystine	0.28	0.25
		Minerals&Vitamins	5.4	6.2
[g%]		[g%]		
Estimated insoluble dietary fibers	22.1	Cellulose	4.7	2
Estimated soluble dietary fibers	3.1	Lignocellulose	0	1
		Pectin	0	6.8
		Inolin	0	6.8
